# Detecting Spread of Avian Influenza A(H7N9) Virus Beyond China

**DOI:** 10.3201/eid2105.141756

**Published:** 2015-05

**Authors:** Alexander J. Millman, Fiona Havers, A. Danielle Iuliano, C. Todd Davis, Borann Sar, Ly Sovann, Savuth Chin, Andrew L. Corwin, Phengta Vongphrachanh, Bounlom Douangngeun, Kim A. Lindblade, Malinee Chittaganpitch, Viriya Kaewthong, James C. Kile, Hien T. Nguyen, Dong V. Pham, Ruben O. Donis, Marc-Alain Widdowson

**Affiliations:** Author affiliations: Centers for Disease Control and Prevention (CDC), Atlanta, Georgia, USA (A.J. Millman, F. Havers, A.D. Iuliano, C.T. Davis, R.C. Donis, M.-A. Widdowson);; CDC, Phnom Penh, Cambodia (B. Sar);; Ministry of Health, Phnom Penh (L. Sovan);; National Institute of Public Health, Phnom Penh (S. Chin);; CDC, Vientiane, Laos (A.L. Corwin);; National Center for Laboratory Epidemiology, Vientiane (P. Vongphrachanh);; Ministry of Agriculture and Forestry, Vientiane (B. Douangngeun);; Thailand Ministry of Public Health–CDC Collaboration, Nonthaburi, Thailand (K.A. Lindblade);; National Institute of Health, Nonthaburi (M. Chittaganpitch);; Ministry of Agriculture and Cooperatives, Bangkok, Thailand (V. Kaewthong); CDC, Hanoi, Vietnam (J.C. Kile);; Ministry of Health, Hanoi (H.T. Nguyen); Ministry of Agriculture and Rural Development, Hanoi (D.V. Pham)

**Keywords:** Influenza, A(H7N9), A(H5N1), surveillance, viruses, China, Vietnam, Cambodia, Laos, Thailand, Southeast Asia, poultry, respiratory infections

## Abstract

During February 2013–March 2015, a total of 602 human cases of low pathogenic avian influenza A(H7N9) were reported; no autochthonous cases were reported outside mainland China. In contrast, since highly pathogenic avian influenza A(H5N1) reemerged during 2003 in China, 784 human cases in 16 countries and poultry outbreaks in 53 countries have been reported. Whether the absence of reported A(H7N9) outside mainland China represents lack of spread or lack of detection remains unclear. We compared epidemiologic and virologic features of A(H5N1) and A(H7N9) and used human and animal influenza surveillance data collected during April 2013–May 2014 from 4 Southeast Asia countries to assess the likelihood that A(H7N9) would have gone undetected during 2014. Surveillance in Vietnam and Cambodia detected human A(H5N1) cases; no A(H7N9) cases were detected in humans or poultry in Southeast Asia. Although we cannot rule out the possible spread of A(H7N9), substantial spread causing severe disease in humans is unlikely.

## Abstract 2

This virus is unlikely to have spread substantially among humans in Vietnam, Thailand, Cambodia, and Laos.

Novel low pathogenic avian influenza (LPAI) A(H7N9) virus emerged in February 2013 and, as of March 3, 2015, a total of 602 laboratory-confirmed human infections, including 227 deaths, had been reported (*1**–**3*). Most human cases have had live poultry or live-bird market (LBM) environmental exposure; person-to-person spread appears infrequent (*4*). However, as circulation of A(H7N9) virus becomes more widespread, the probability increases for mutations enabling efficient person-to-person transmission.

Similar fears accompanied the reemergence in 2003 of highly pathogenic avian influenza (HPAI) A(H5N1) virus, which caused ≈45 human cases in Vietnam and Thailand within 12 months. As of March 3, 2015, A(H5N1) virus had resulted in 784 human cases, including 429 deaths, in 16 countries and poultry outbreaks in 53 countries (*3*,*5*–*7*). In response to these outbreaks, avian influenza surveillance systems were created to monitor A(H5N1) activity and to detect other novel influenza viruses.

In contrast to the rapid international spread of A(H5N1) virus in poultry and humans within 12 months after its reemergence, no autochthonous A(H7N9) cases in animals or humans have been reported outside mainland China (*3*), despite a higher incidence of reported A(H7N9) than A(H5N1) cases in humans; A(H7N9) detection in poultry and humans; and the presence of the virus in border provinces in southern, western, and northeastern China (*2*,*8*,*9*). Surveillance systems in Southeast Asia have detected new A(H5N1) cases in humans and poultry since the A(H7N9) virus was first identified (*3*). Whether the absence of reported A(H7N9) among humans or poultry outside mainland China represents a lack of spread or whether regional surveillance systems are insufficiently sensitive to detect A(H7N9) remains unclear. Because A(H5N1) virology and epidemiology differ from those of A(H7N9), assessing how A(H7N9) might spread and whether surveillance would detect it remains critical for countries to prepare control measures and to monitor virologic and epidemiologic changes.

We highlight differences and similarities between A(H5N1) and A(H7N9) viruses and hypothesize scenarios related to possible A(H7N9) virus spread. Then we describe human and animal influenza surveillance data from 4 Southeast Asian countries where A(H5N1) has been detected—Vietnam, Cambodia, Laos, and Thailand—to assess the likelihood that surveillance systems designed for A(H5N1) would have detected human or animal A(H7N9) infections during the predominant months of A(H7N9) virus circulation during 2014.

## Comparison of A(H5N1) and A(H7N9) Epidemiology and Virology

A(H5N1) and A(H7N9) have epidemiologic and virologic similarities and differences. These features have implications for detection by existing human and animal surveillance systems (Table 1).

**Table 1:** Characteristics of influenza A(H5N1) and A(H7N9) infection and implications for surveillance system detection of A(H7N9) in humans and animals*

Characteristic	A(H5N1)	A(H7N9)	Reference	Surveillance system implications for A(H7N9) detection
Clinical signs and symptoms	Fever, cough, pneumonia, respiratory failure	Fever, cough, pneumonia, respiratory failure	(*1,4*)	SARI and ILI surveillance systems should detect with equal efficacy
Disease severity	Critical and fatal (60%)	Most are critical; mild infections reported in children	(*4*)	Hospital-based platforms would be most likely to detect cases
Patient age, y	<20	>60	(*4*)	Surveillance systems that do not cover older adults may not detect case
Seasonality	December–March (average)	Most cases in 2nd wave occurred December–March 2013–2014	(*3*)	Surveillance will be more likely to detect a case when the virus in circulating; however, additional data are needed to establish the seasonality of A(H7N9)
Geography	Primarily rural (farm)	Primarily urban (LBM)	(*4*)	Surveillance systems that do not cover visitors to LBMs may be unable to detect cases
Transmissibility from poultry or environment to humans	Appears low	Appears moderate	(*10*)	Surveillance systems should assess for poultry or environmental exposures, and known exposures should prompt testing in suspected cases of avian influenza
Person-to-person transmission	Appears uncommon	Appears uncommon	(*1,11*)	Surveillance systems will probably detect sporadic cases that have identifiable poultry exposures
History of poultry exposure	Common	Common	(*4*)	Animal surveillance is critical for detection in poultry and assisting with targeting control measures
Pathogenicity in chickens	High	Low	(*5,11*)	Infection with A(H7N9) does not appear to cause disease in poultry. Surveillance for detecting A(H7N9) in poultry requires targeted risk assessment and active testing.
Effects in wild bird species	Detected in wild bird species	Limited data	(*11–13*)	Poultry surveillance directed at either back-yard farms or commercial poultry farms (depending on prevalence) and LBMs should be sufficient to detect cases

*ILI, influenza-like illness; LBM, live-bird market; SARI, severe acute respiratory infection.

### Clinical Presentation and Demographics

Reported A(H5N1) and A(H7N9) infections in humans generally present as severe respiratory disease with fever, cough, and pneumonia, often leading to respiratory failure (*1*,*4*). Most A(H5N1) cases in Asia and A(H7N9) cases in China are detected in hospitals; therefore, existing hospital-based surveillance systems should detect severe infections of both viruses. However, hospital-based surveillance alone might be unlikely to detect mild cases or cases outside surveillance areas. Most A(H5N1) cases were reported in children and young adults (median age ≈17 years). In contrast, the median age of persons with A(H7N9) is 58 years; mild cases have been reported predominantly in children (*4*). Therefore, surveillance systems biased toward younger patients are less likely to detect A(H7N9) cases.

### Seasonality

Most A(H5N1) infections in humans and poultry occur during November–May in China and Southeast Asia (*3*,*14*); similarly, most A(H7N9) cases occurred in February–May 2013 and December 2013–March 2014. Although A(H7N9) seasonality data are limited, A(H7N9) infections probably would increase during December–March; however, sporadic cases might be detected in other months.

### Host Range and Pathogenicity in Animals

These viruses have some genetic similarities predicted to alter their adaptability to animal hosts. Many A(H5N1) and most A(H7N9) viruses have internal genes derived from LPAI A(H9N2) viruses, which might confer adaption to poultry (*12*). Additionally, internal genes shared by many of these viruses have several mutations demonstrated to enhance adaptation to mammalian hosts (PB2 E672K, PB1-F2, M1 N30D and T215A, and NS1 P42S). These and other shared mutations might affect viral adaptation to mammalian hosts and might explain similarly severe clinical infections in humans (*12*,*15*,*16*). These viruses currently have differences in host range and pathogenicity in animals that could affect surveillance. A(H5N1) circulates among domestic chickens, ducks, geese, and other poultry; sporadic outbreaks occur in wild migratory waterfowl species (*5*,*17*). A(H7N9) has been detected primarily in chickens and infrequently in ducks, pigeons, and quail (*10*,*18*–*20*). A(H7N9) has not been detected among wild waterfowl. Experimentally, A(H7N9) viruses replicate well in poultry, quail, and Muscovy ducks but are less infectious and result in decreased shedding in other wild bird species, which may limit their ability to spread in these species (*10*). This feature may relate in part to a neuraminidase stalk deletion in A(H7N9), which is considered a marker of adaptation to poultry rather than to wild waterfowl (*21*); because similar neuraminidase stalk deletions have been documented in A(H5N1) viruses isolated from wild birds, the implications of this feature remain unclear (*22*).

A(H5N1) viruses with the A/goose/Guangdong/1/1996-like hemagglutinin gene are classified as HPAI viruses because infections in chickens or other gallinaceous species cause high-level replication throughout many tissues, extensive shedding/environmental contamination, and generally high death rates (*5*,*8*,*11*). In contrast, A(H7N9) viruses are classified as LPAI because poultry infections cause low-level viral shedding and replication is limited to digestive and respiratory tracts; infected poultry typically remain asymptomatic. Nevertheless, some LPAI viruses, such as A(H9N2), have transmitted extensively in poultry in Asia (*23*), possibly because they have increased environmental stability relative to HPAI viruses (*24*). Poultry illnesses and deaths alert health authorities about possible A(H5N1) outbreaks and trigger enhanced surveillance in humans (*5*). Conversely, the low pathogenic nature of A(H7N9) means that passive surveillance cannot rely on the same triggers used for A(H5N1). To detect A(H7N9), systematic, risk-based surveillance and sampling of asymptomatic poultry is more appropriate.

### Poultry Exposure and Transmissibility to Humans

Backyard poultry are a source for exposure to A(H5N1) virus, but this exposure has been reported less frequently among A(H7N9)-infected persons, whereas exposure to chickens (usually slower-growing yellow chickens or Silkie chickens) or environmental exposure in LBMs are the major risk factors for human A(H7N9) infection (*4*,*14*,*25*). Despite extensive testing reported by Chinese authorities, few A(H7N9)-positive samples have been detected in commercial farms (*9*). The sources of virus exposure to humans may change if A(H7N9) spreads further among backyard poultry and large commercial farms. Although poultry exposure is a risk factor for both viruses, A(H7N9) may be more transmissible from infected poultry or poultry environments to humans because it has a glutamine to leucine amino acid substitution at position 217 (position 226 in H3 numbering), whereas A(H5N1) virus maintains a more strictly conserved glutamine (avian consensus) at the equivalent H5 hemagglutinin position (*10*). This substitution confers a higher virus specificity to α2,6 sialic acid receptors (which predominates on human respiratory epithelial cells), possibly explaining the high incidence of human A(H7N9) cases (*10*).

## Potential Spread of A(H7N9)

**Figure 1:**
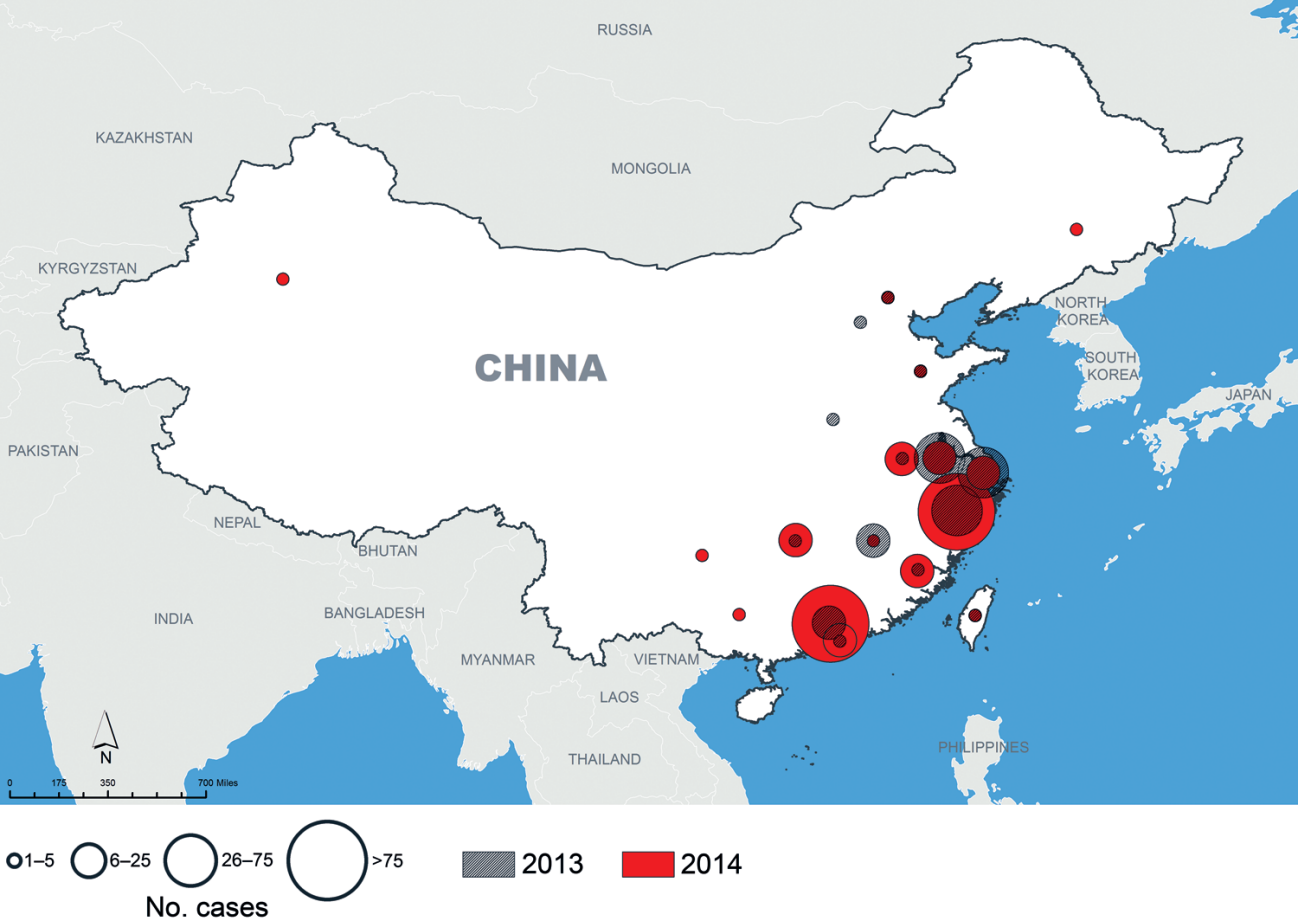
Avian influenza A(H7N9) in humans, China, 2013–2014. Data were obtained from the World Health Organization as reported from the National Health and Family Planning Commission (http://www.who.int/influenza/human_animal_interface/influenza_h7n9/en/).

Since A(H7N9) emerged, 2 complete waves of infections have occurred; the second wave is defined as cases occurring during October 1, 2013–September 30, 2014 and affecting mostly the southeastern provinces of China (Figure 1). A third (ongoing) wave is defined as cases since October 1, 2014. A(H7N9) virus does not transmit easily between humans, and person-to-person spread has been limited to 2 or possibly 3 generations of transmission (*2*,*4*). Assuming transmission remains unchanged, geographic spread probably will occur through travel of infected humans or infected poultry. Several persons exposed to A(H7N9) virus in China traveled to Hong Kong, Taiwan, Malaysia, and Canada; became ill; and were deemed to have imported infections (*2*). Additional sporadic A(H7N9) infections might occur in travelers, but appropriate isolation measures should prevent further spread (*2*).

**Figure 2:**
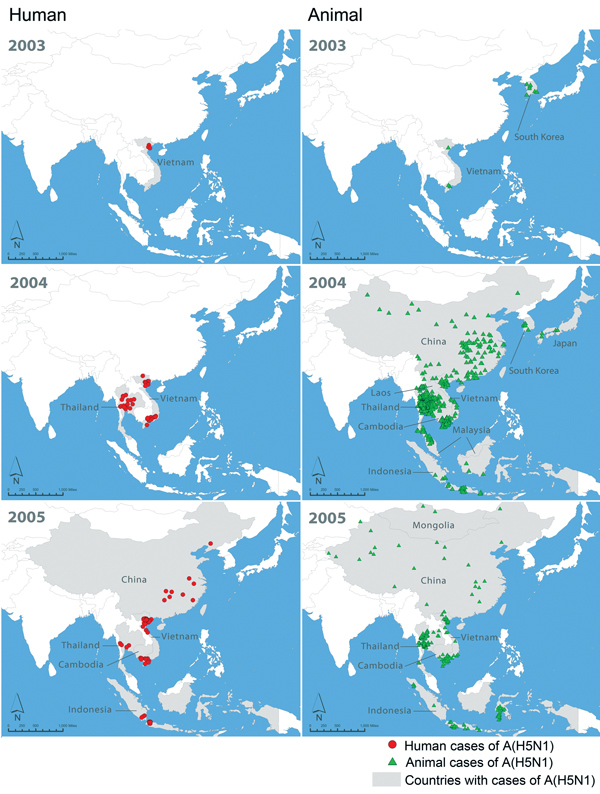
Initial 2-year spread of human cases and poultry outbreaks of influenza A(H5N1) in China and Southeast Asia, December 2003–2005. Data on A(H5N1) in humans were obtained from the World Health Organization (http://www.who.int/influenza/human_animal_interface/en/). Data on outbreaks of A(H5N1) in poultry were obtained from the World Organisation for Animal Health (outbreaks before 2005 from http://www.oie.int/en/animal-health-in-the-world/the-world-animal-health-information-system/data-before-2005-handistatus/; outbreaks after 2005 from http://www.oie.int/wahis_2/public/wahid.php/Wahidhome/Home).

LBMs create environments that can amplify avian influenzas viruses and increase risk of human infection (*5*,*19*,*26*). Large informal poultry movements between China and Southeast Asian countries also pose a risk for spread (*8*,*27*). The first confirmed human A(H5N1) case outside China occurred in December 2003 in Vietnam and foreshadowed the virus’ rapid regional spread in humans and poultry (Figure 2). Phylogeographic studies suggest that A(H5N1) virus was introduced to Vietnam from China through these poultry trade routes; A(H7N9) virus is similarly likely to be introduced in domestic poultry in Vietnam (*27*,*28*). However, because A(H7N9) preferentially infects different poultry species than A(H5N1), different poultry value chains might be implicated in this potential spread.

Wild migratory birds have contributed to spread of A(H5N1) virus along regional flyways (*29*,*30*). A(H7N9) virus was detected in a nonmigratory wild sparrow in China during spring 2013 but has not been identified in other wild bird species (*13*). This finding suggests that A(H7N9) infection is not widespread in wild birds, and the possible risk for regional spread by wild birds is currently low.

## Surveillance for Avian Influenza in Southeast Asia

**Figure 3:**
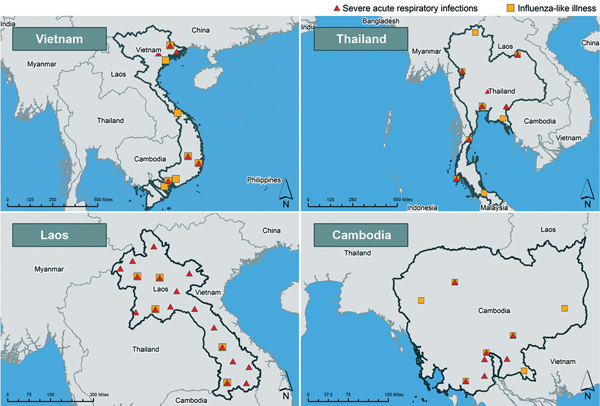
Severe acute respiratory infection (SARI) and influenza-like Illness (ILI) sentinel sites in Vietnam, Thailand, Laos, and Cambodia. A given location might have >1 SARI or ILI sentinel site. SARI sites in Laos include planned SARI sites and 8 nonsentinel SARI sites. (The Lang Son and Quang Ninh province sites continue to operate. The Hanoi site operated until June 2014.)

Vietnam, Thailand, Laos, and Cambodia each operate at least 2 national systems for influenza surveillance in humans: 1) sentinel inpatient-based severe acute respiratory infection (SARI) and 2) sentinel outpatient-based influenza-like illness (ILI). Additionally, most operate event-based or passive surveillance systems for pneumonia (Figure 3) with prespecified case definitions (Technical Appendix Table). World Health Organization member states must report all cases of A(H5N1) and A(H7N9) in humans as required by the International Health Regulations (2005); the World Organization for Animal Health mandates reporting of outbreaks of HPAI in birds by the Terrestrial Animal Health Code (*31*,*32*).

### Vietnam

#### Human Surveillance

Since May 2013, four regional public health institutes in Vietnam have had real-time reverse transcription PCR (rRT-PCR) A(H7N9) testing capacity, including the institutes housing the 2 National Influenza Centers (NICs)—the National Institute of Hygiene and Epidemiology in Hanoi and the Pasteur Institute in Ho Chi Minh City (*33*). Vietnam conducts sentinel SARI surveillance at 8 sites and ILI surveillance at 10 sites. Four SARI sites have operated continuously since April 2013. Another 4 were established in December 2013, including 3 in Lang Son and Quang Ninh provinces, which are entry points for Chinese poultry (*34*). Of ≈2,500 SARI specimens tested for influenza by using Centers for Disease Control and Prevention (Atlanta, GA, USA) testing protocols during April 1, 2013–May 30, 2014, none were positive for A(H5N1) or A(H7N9) virus. During the same period, no ILI specimens tested positive for A(H7N9); 1 was A(H5N1) positive.

Since 2006, Vietnam has operated a nationwide passive surveillance system for pneumonia in all hospitals (*35*). This surveillance system identified 33 human A(H5N1) virus infections, but no A(H7N9) virus infections have been detected since testing for this virus began in December 2013 (*35*). The median age of patients in this system was 43 years. Table 2 shows results from all 3 systems during April 1, 2013–May 30, 2014.

**Table 2:** Surveillance for SARI and ILI and passive surveillance for pneumonia for avian influenza in humans, 4 Southeast Asia countries, April 1, 2013–May 30, 2014*

Surveillance system	No. illnesses meeting case definition	Total no. samples tested	A(H5N1), no. positive/no. tested	A(H7N9), no. positive/no. tested
Vietnam				
SARI	11,558	2,485	0/798	0/798
ILI	29,027	3,770	0/0	0/0
Passive surveillance for pneumonia†	238	237	4/70	0/70
Thailand				
SARI	‡	1,025	0/106§	0/106§
ILI	‡	3,850	0/807	0/807
Passive surveillance for pneumonia	‡	157	0/157	0/43
Event-based surveillance	18 outbreaks	162	0/69§	0/69§
Surveillance for severe or fatal pneumonia	208	208	0/14§	0/14§
Cambodia				
SARI	2,282	2,282	7/219	0/0
ILI	1,567	1,567	1/10	0/0
Laos				
SARI	1,469	698	0/15	0/15
ILI	8,962	1,550	0/0	0/0

*ILI, influenza-like illness; SARI, severe acute respiratory infection. †Severe viral pneumonia surveillance system. ‡Total number of illnesses meeting the case definition is unknown for ILI. For SARI, the number of illnesses meeting the case definition is unknown but believed to be close to 100% of persons sampled. Reporting to the passive pneumonia surveillance system is in accordance with physician discretion, and no clear case definition is applied. §A(H5N1) and A(H7N9) testing is conducted only on specimens positive for influenza A virus.

#### Animal Surveillance

The Vietnam Ministry of Agriculture and Rural Development, Department of Animal Health, National Center for Veterinary Diagnosis, obtained A(H7N9) laboratory testing capacity in June 2013. During December 5, 2013–March 6, 2014, the Department of Animal Health conducted active weekly surveillance for A(H7N9) and A(H5N1) using rRT-PCR in 13 traditional and nontraditional LBMs in Hanoi, Quang Ninh, and Lang Son provinces, areas historically known to contain markets selling smuggled poultry. No A(H7N9) virus was detected from 737 poultry oropharyngeal and cloacal specimens and 555 poultry cage fecal and water samples. Additionally, the Ministry of Agriculture and Rural Development conducted biweekly surveillance in 60 LBMs in 9 northern provinces bordering China (*36*). None of the 25,000 samples had tested positive for A(H7N9) as of August 2014 (*36*). Routine A(H7N9) surveillance is not performed on poultry farms.

### Thailand

#### Human Surveillance

In April 2013, the Thai NIC in Nonthaburi acquired A(H7N9) testing capacity and has since trained 14 regional laboratories (*33*). SARI sentinel surveillance is conducted in hospitalized patients at 7 sites in 7 provinces; 2 were established after A(H7N9) emerged. ILI surveillance is conducted at 10 sites in 9 provinces, where 10 outpatients with ILI are sampled weekly for influenza testing.

Thailand operates 3 additional influenza surveillance systems: a national passive system for pneumonia in public outpatient and inpatient facilities; a national event-based system for reporting clusters of severe respiratory disease or respiratory illnesses associated with dead or dying poultry; and a severe and fatal pneumonia sentinel system in 30 hospitals nationwide. In the passive pneumonia system, physicians send samples at their discretion. During April 1, 2013–May 30, 2014, no human cases of A(H7N9) or A(H5N1) were detected (Table 2). Among patients tested for A(H7N9) virus, the median ages were 29 years (pneumonia surveillance system) and 31 years (event-based system). Of 208 cases identified by the severe or fatal pneumonia system during July 2013–March 2014 (≈70% <5 years old), 14 were influenza A positive and none A(H7N9) positive.

#### Animal Surveillance

Poultry production in Thailand has strict biosecurity practices (*5*). Moreover, Thailand is a net poultry exporter and shares no borders with China. However, informal backyard poultry trade for LBMs at its Laos and Cambodia borders may present an indirect importation risk.

The National Institute of Animal Health in Bangkok and Regional Veterinary Research and Development Centers perform diagnostic testing for avian influenza viruses, including A(H7N9). Active surveillance is conducted routinely in poultry farms in 77 provinces; viral isolation is performed on pooled samples of cloacal swabs (5 poultry per pool). During April 1, 2013–May 30, 2014, a total of 8,829 pooled samples from poultry farms were tested, and neither A(H5N1) nor A(H7N9) was detected.

Active poultry and environmental surveillance was conducted in 9 Bangkok LBMs during November 2013. A total of 1,853 samples consisting of 1,619 oropharyngeal or fecal swabs and poultry serum and 234 environmental water samples were tested for A(H7N9) by RT-PCR; none tested positive.

### Cambodia

#### Human Surveillance

In Cambodia, testing capacity for A(H7N9) virus was available in September 2013 at the national laboratory network. The network comprises the Institute Pasteur; a World Health Organization NIC; and the National Institute Public Health Laboratory (*33*).

The Cambodia Ministry of Health operates 8 SARI sentinel sites in Phnom Penh and 6 provinces. Four have operated since 2009; 4 sites were added in provinces bordering Vietnam in 2013. Seven ILI sentinel sites operate in Phnom Penh and 6 additional provinces. During April 1, 2013–May 30, 2014, eight A(H5N1) cases and no A(H7N9) cases were detected in humans in Cambodia (Table 2).

The Ministry of Health also conducts event-based surveillance through CAM-EWARN (Cambodia Early Warning and Response Network) (*37*). CAM-EWARN operates in 1,199 sites ranging from specialty hospitals to health centers in all provinces and relies on voluntary reporting. Of 26 A(H5N1) cases in humans during 2013, 21 were reported through CAM-EWARN because these patients sought care at nonsentinel sites. Most cases reported through this system were in children <5 years of age. No human A(H7N9) cases have been reported through CAM-EWARN.

#### Animal Surveillance

The National Animal Research Institute and Institute Pasteur conducted poultry and environmental surveillance for A(H7N9) in 4 LBMs in 4 provinces during 2013. During this time, rRT-PCR was performed on 528 poultry throat and cloacal samples and 792 environmental samples; all were negative for A(H7N9). Routine A(H7N9) surveillance is not performed on poultry farms.

### Laos

#### Human Surveillance

Since May 2013, the Laos NIC based in the National Center for Laboratory and Epidemiology has had testing capacity for A(H7N9) virus (*33*). The Hospital Sentinel Virological Surveillance Network comprises SARI surveillance in 7 sentinel sites in 5 provinces and ILI surveillance at 8 sites in 5 provinces. The SARI surveillance platform is expanding and will eventually comprise 26 sentinel sites in all provinces (Figure 3). During April 1, 2013–May 30, 2014, fifteen specimens from SARI patients were tested for A(H5N1) and A(H7N9), and none were positive; no specimens from ILI surveillance were tested for A(H5N1) or A(H7N9) virus (Table 2).

#### Animal Surveillance

The National Animal Health Laboratory (NAHL) acquired animal A(H7N9) testing capacity in April 2013. NAHL conducts routine surveillance in 4 LBMs in 3 provinces bordering China. A total of 892 poultry oropharyngeal specimens and 74 environmental specimens were negative for A(H7N9) virus by RT-PCR. An additional 892 poultry serum samples were negative for H7 antibodies. During April 1, 2013–May 30, 2014, NAHL collected 1,666 poultry swab samples and 137 environmental samples from poultry farms and villages in 3 provinces bordering China; all tested negative for A(H5N1) and A(H7N9).

## Conclusions

A(H7N9) and A(H5N1) viruses can cause severe disease in humans, do not transmit easily from person to person, and are primarily linked to exposure to infected poultry or contaminated environments. However, several major differences—including older age of human A(H7N9) patients, greater risk for transmission from infected poultry to humans, and lower pathogenicity of A(H7N9) virus infection in poultry—have implications for A(H7N9) virus detection. A(H7N9) will circulate in poultry without the typical HPAI-associated morbidity and mortality and can spread undetected, making A(H7N9) passive surveillance and control in poultry species challenging. Systematic, risk-based poultry surveillance is appropriate but might still miss A(H7N9) cases. As probably occurred with A(H5N1), A(H7N9) may spread through informal poultry trade between China and neighboring countries; however, the predilection of A(H7N9) for certain poultry species may favor different poultry value chains. This spread may have already occurred; although surveillance of poultry provides useful monitoring, it is unlikely to detect A(H7N9)-infected poultry until the virus becomes widespread because large sample sizes are needed to detect cases in a low-prevalence poultry population. Detection might not occur in poultry until a human case is identified, which would trigger additional poultry surveillance; notably, however, targeted poultry surveillance activities at known sites of exposure in China have had few detections despite extensive testing (*20*). The initial emergence of A(H5N1) in Hong Kong in 1997 was, like that of A(H7N9), associated with LBMs, but the virus has since become established in all poultry sectors, including endemicity in backyard poultry and outbreaks in wild birds (*34*). A(H7N9) virus might do the same, and its epidemiologic profile might change. However, the mean age of A(H5N1)-infected persons in 1997 was <10 years; no A(H5N1) cases have been reported in China in persons >65 years of age, which suggests that exposure alone cannot explain the age differences between persons with A(H5N1) and A(H7N9) infections.

Although we cannot rule out the possibility of human A(H7N9) disease in Southeast Asia, substantial A(H7N9) spread resulting in the widespread occurrence of severe infections in humans is unlikely to have occurred in Vietnam, Thailand, Cambodia, and Laos. All 4 countries have the laboratory capacity to detect A(H7N9) and well-developed hospital-based surveillance systems. Moreover, Vietnam, Thailand, and Cambodia operate passive pneumonia surveillance systems covering wide geographic areas and include public and private health care facilities. Since 2006, when Vietnam established passive surveillance, the system has been critical to detecting A(H5N1) in humans. Similarly, of 26 human A(H5N1) cases detected by CAM-EWARN in 2013, 18 sought care at private health care facilities and would not have been detected by sentinel platforms. Vietnam and Cambodia have detected A(H5N1) in humans since 2013, suggesting that severe A(H7N9) infections in humans have not occurred because either the virus has not spread to these countries or it has a lower incidence than A(H5N1). Sporadic traveler-associated A(H7N9) might be detected, but if person-to-person transmission remains limited, this mechanism should not contribute to continued spread of A(H7N9) in humans.

Although sentinel platforms in all countries may be adequate for detecting A(H7N9), their limited geographic coverage could miss cases. Efforts to expand these platforms may enhance case detection capabilities, especially in Laos, which relies solely on its sentinel surveillance systems. Therefore, robust passive reporting systems may be important to detect severe A(H7N9) cases early. Additionally, although these sentinel- and nonsentinel-based systems have performed well for A(H5N1), most detected cases occurred in children or young adults in Vietnam and Thailand or came from 1 pediatric hospital in Cambodia. Most surveillance systems cover all ages and should be able to detect illness meeting the case definition; gaps in age coverage (i.e., surveillance only at pediatric hospitals) would result in decreased sensitivity for detecting A(H7N9) because it is more likely to cause severe disease in persons >60 years of age.

Our analysis has limitations. We were unable to evaluate systems in other Asia countries; of particular concern is Myanmar, which has reported outbreaks of A(H5N1) and shares a long border with China across which it imports large quantities of poultry (*3*). Additionally, since July 2014, A(H7N9) has been detected in humans in Xinjiang Province in China (*38*), which borders 8 countries. A(H7N9) circulation in China’s western region suggests that it is probably more geographically widespread than previously realized. Additionally, our analysis focused on surveillance programs operated by governments in Vietnam, Thailand, Cambodia, and Laos. Additional surveillance may exist for avian influenza, particularly through research studies and other entities our analysis does not cover.

Since 2013, we have observed the detection of multiple novel avian influenza viruses, including A(H7N9), A(H6N1), A(H5N6), and A(H10N8) (*39*,*40*). On the basis of our assessment, we believe substantial spread of A(H7N9) virus resulting in severe infections in humans is unlikely to have occurred in Vietnam, Thailand, Cambodia, or Laos. Given the virus’ characteristics, there likely will be no obvious signal if it spreads to Southeast Asia. Well-designed, routine surveillance and astute clinicians are essential for detecting the first case beyond China. The experience with A(H5N1) shows how countries in Southeast Asia designed systems capable of detecting and responding to avian influenza in poultry and humans, but vulnerabilities remain. Growing trade networks and economic integration mean weaknesses in individual surveillance systems can leave the entire region vulnerable. Governments must be vigilant against new and reemerging disease threats by rapidly responding to suspected outbreaks in animals and humans, educating health care professionals and the public, and working with partners to enhance animal health and public health systems. 

**Technical Appendix.** Case definitions of severe acute respiratory infection, influenza-like illness, and passive pneumonia surveillance systems, Vietnam, Thailand, Cambodia, and Laos.
