# Supplementary material for: Detecting Spread of Avian Influenza A(H7N9) Virus Beyond China
Source: Emerg Infect Dis. 2015 May;21(5):741–9. doi: 10.3201/eid2105.141756 (PMC4412232; doi:10.3201/eid2105.141756)
Supplement: Supplementary file 1 — Technical Appendix. Case definitions of severe acute respiratory infection, influenza-like illness, and passive pneumonia surveillance systems, Vietnam, Thailand, Cambodia, and Laos. [file 14-1756-Techapp-s1.pdf]

Article DOI: http://dx.doi.org/10.3201/eid2105.141756

Detecting Spread of Influenza A(H7N9) Virus Beyond China

Technical Appendix

Technical Appendix Table. Case definitions of severe acute respiratory infection (SARI), influenza-like illness (ILI), and passive pneumonia surveillance systems, Vietnam, Thailand Cambodia, and Laos

Country/surveillance system	Case definition
Vietnam	
SARI	(Onset within 7 d)<br>For ≥5 yrs old<br>• Temperature ≥38.0ºC AND<br>• Cough OR Sore throat AND<br>• Shortness of breath OR difficult breathing AND<br>• Requires hospitalization<br>For <5 yrs old:<br>• Temperature ≥38.0ºC AND<br>• Cough or difficult breathing AND<br>• At least one of the following<br>- Unable to drink or breastfeed at all<br>- Vomits everything (not only occasional)<br>- Chest indrawing (retractions under ribcage on inspiration)<br>- Lethargic or unconscious (ensure patient is awake)<br>- Oxygen saturation < 90%<br>- Nasal flaring<br>- Convulsions<br>- Stridor in a calm child<br>- Grunting<br>- Tachypnea (<2 mo—RR >60; 2–12 mos—RR >50; 1–4yrs—RR >40) AND<br>• Requires hospitalization
ILI	• Temperature >38°C (temperature measurement is required at consultation room)<br>• Cough and/or sore throat<br>• No other causes are identified
Passive pneumonia surveillance	• Temperature >38°C (measured at consultation room or reported)<br>• Difficult breathing (determined by rapid respiratory rate, shortness of breath or blood oxygen/ carbon dioxide saturation level)<br>• Chest radiograph consistent with viral pneumonia<br>• No suspect diagnoses of other etiology, such as bacterial pneumonia or tuberculosis were made.
Thailand	
SARI	• Clinical diagnosis of pneumonia or community-acquired pneumonia or ILI
ILI	• Temperature ≥ 38°C or having fever history AND<br>• Cough and/or sore throat during the past 5 d
Passive pneumonia surveillance	• Clinician defined
Event-based surveillance	• Cluster of ≥2 cases linked in time and space; severe respiratory illness associated with dead or dying poultry
Severe and fatal pneumonia surveillance	• Community-acquired pneumonia requiring intubation that does not respond to treatment within 48 hours or resulted in death
Cambodia	
SARI	• Temperature >38°C AND<br>• Cough or sore throat AND<br>• Shortness breath or difficult breathing AND<br>• Admission to the hospital, with onset disease within 10 d.
ILI	• Temperature> 38°C AND<br>• Cough or sore throat with onset disease within 5 d
CAM-EWARN	• A person >5 y old with a temperature ≥3°8C and any following: cough or difficult breathing or shortness of breath.<br>• A child 1–5 y old with cough or difficult breathing AND breathing rate of >40/min.

Country/surveillance system	Case definition
	• An infant aged 2 mos to <1 y with cough or difficulty breathing AND breathing rate of >50/mi.
	• Any person with evidence of pneumonia on chest radiograph.
Laos	
SARI	• An acute respiratory infection with history of fever or measured temperature ≥38°C AND
	• cough AND
	• onset within the last 7 d AND
	• requires hospitalization
ILI	• An acute respiratory infection with measured temperature of ≥38°C AND
	• cough WITH onset within the last 7 d
